# Ertapenem-Induced Thrombocytosis

**DOI:** 10.7759/cureus.1263

**Published:** 2017-05-19

**Authors:** Ramon A Docobo, Sumera Bukhari, Zulfiqar Qutrio Baloch

**Affiliations:** 1 Medicine, Brandon Regional Hospital; 2 Internal Medicine, Seton Hall University-St. Francis Medical Center, Trenton, NJ; 3 Internal Medicine, Brandon Regional Hospital

**Keywords:** ertapenem, thrombocytosis, beta-lactam

## Abstract

Ertapenem is a β-lactam antibiotic that has a broad spectrum of anti-microbial coverage. Hematological adverse events like thrombocytosis, neutropenia, and neutropenia are infrequent. Here we report a rare case of drug-induced thrombocytosis in a 68-year-old female, who was treated with ertapenem for the diagnosis of complicated abdominal infection. This case emphasizes that any patient with thrombocytosis should be assessed with a careful and detailed history with consideration for possible drug side effects.

## Introduction

Carbapenems are parenteral bactericidal β-lactam antibiotics that have an extremely broad spectrum anti-microbial coverage [1-2]. Carbapenems (imipenem, meropenem, doripenem, and ertapenem) are an important class of antibiotics for infections due to community-acquired or nosocomial aerobic and anaerobic organisms, especially the extended spectrum beta-lactamase (ESBL) producing Enterobacteriaceae. Ertapenem is advantageous in its extended half-life, allowing once daily dosing. It has greater plasma-protein binding properties when compared to other carbapenems [1-2]. It is useful for the treatment of complicated intra-abdominal, complicated skin/skin structure, and acute pelvic infections as well as complicated urinary tract infections (UTIs) and community-acquired pneumonia (CAP) [1-2]. The most common clinical side effects are diarrhea, infusion site reaction, nausea, and headache. Ertapenem may also cause a mild increase in alanine aminotransferase (ALT), aspartate transaminase (AST), and alkaline phosphatase. Rarely, it can cause hematological changes leading to thrombocytosis, eosinophilia, and neutropenia [3]. Here we report a case of drug-induced thrombocytosis (DIT) as a rare adverse effect of ertapenem therapy in a 68-year-old female with the diagnosis of complicated abdominal infection.

## Case presentation

**Figure 1:**
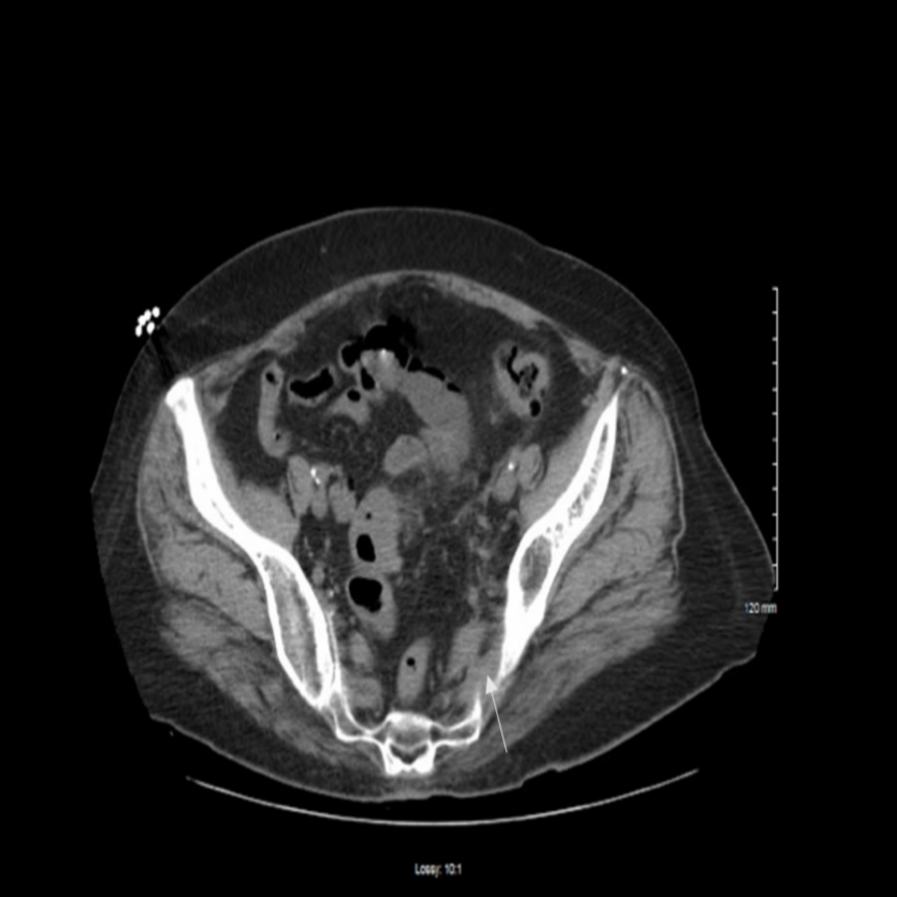
Computerized tomography of abdomen and pelvis. The arrow pointing to the colon wall thickness, recto-sigmoid diverticulitis.

A 68-year-old female with past medical history of diverticulosis, hypertension, diabetes mellitus type 2, and peripheral artery disease presented with near syncopal episode and abdominal pain. She had one episode of diverticulitis before this event. Her past surgical history was significant for a right carotid endarterectomy, appendectomy, hysterectomy, and cholecystectomy. The patient was non-compliant and was not taking any medication for her diabetes, hyperlipidemia, or hypertension. Family history was non-contributory to present condition with no history of blood disorders. She was a chronic smoker with a 35 pack-year smoking history. Social history was remarkable for beer and wine consumption of 3-4 times per week and no recreational drug use. On physical examination: temperature 36.1°F, blood pressure 156/70 mmHg, pulse rate 85 beats per minute, respiratory rate 16 breaths per minute. No orthostatic changes in vital signs were noted. The abdomen was soft, tenderness was noted in left lower quadrant and periumbilical region. Bowel sounds were audible. Neurological exam was normal with no sensory or motor deficits noted. The cardiac exam was unremarkable with normal electrocardiogram (EKG) findings. In the emergency room, computed tomography (CT) of the brain was negative for intra-cerebral bleed. CT of abdomen and pelvis (Figure 1) was done, which showed colon wall thickening at the recto-sigmoid region and mild stranding of the pericolic fat, suggestive of recto-sigmoid diverticulitis.

**Figure 2:**
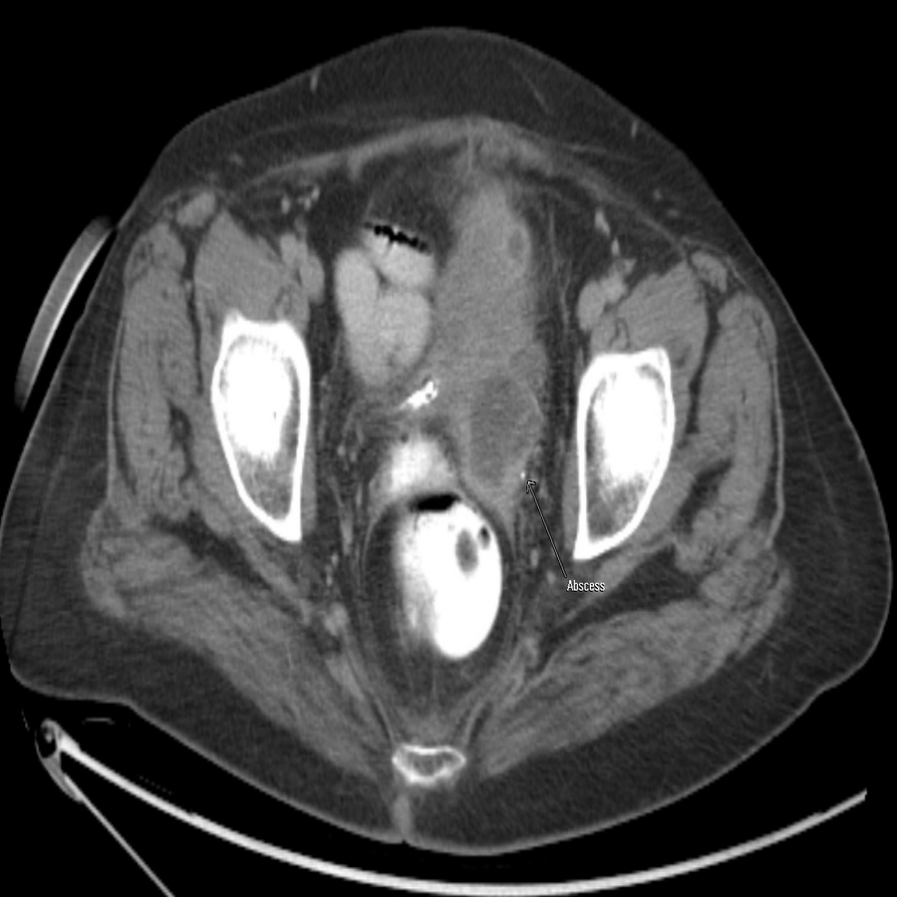
Repeated computerized tomography scan of abdomen and pelvis showing the abscess (arrow).

Subsequently, the patient was admitted for the management of diverticulitis and further evaluation of near-syncope to the telemetry floor. The patient was started on intravenous (IV) ciprofloxacin 400 mg twice a day and metronidazole 500 mg three times in a day. Initial blood workup showed hemoglobin (Hb) of 11g/dL, white blood cells (WBCs) of 20,000/mm^3^ and platelet count of 487,000/mm^3^. The patient's WBC count continued to trend up in next 48 hours, while platelets were 484,000/mm^3^. Infectious diseases recommended switching antibiotics to IV ertapenem due to persistence leukocytosis. A repeat CT scan of abdomen and pelvis (Figure 2) was done, showing peri-diverticular abscess.

**Figure 3:**
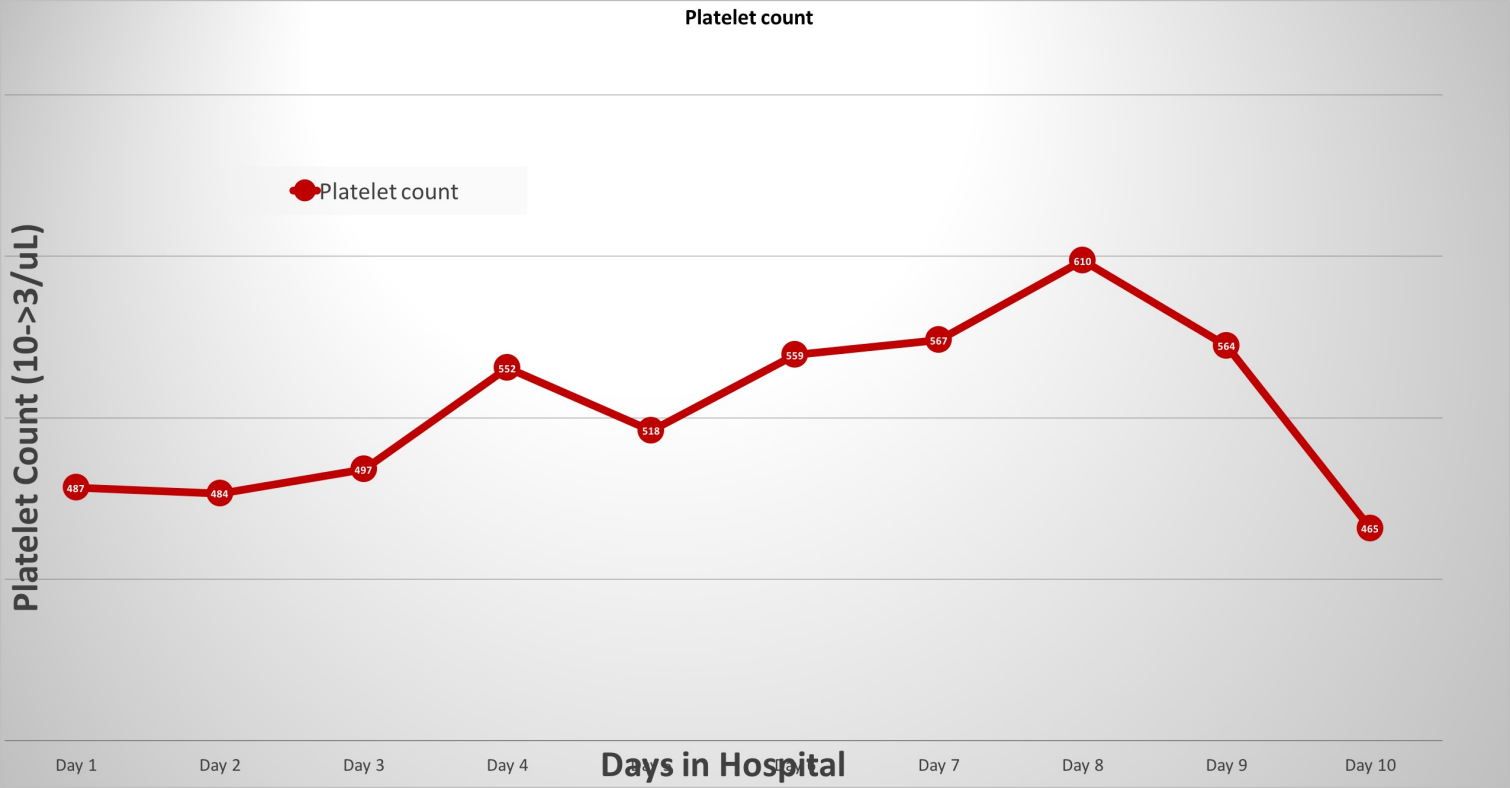
Platelet trend during inpatient course. Ertapenem started on day 4/discontinued on day 9.

CT guided drainage was performed but was unsuccessful as there was not enough fluid to be drained. Over the next several days the patient's WBC count trended down, but platelets kept trending upward and reached to the value of 610,000/mm^3^. Initially, thrombocytosis was thought to be merely an acute phase reaction but the rising trend of platelets, while the patient was improving clinically on antibiotics, was suspicious. The patient was vigilantly observed for possible increased risk of bleeding or thrombotic event during the thrombocytosis period, and other possible causes were ruled out. The ertapenem-induced thrombocytosis was the only possible explanation after reviewing the sequence of events. The concerns were discussed with infectious disease team, and it was recommended to switch antibiotic to oral ciprofloxacin and metronidazole as the patient’s symptoms were improving and she was able to tolerate an oral diet. The patient was observed for next 24 hours while ertapenem was discontinued. At the time of discharge, WBC count was 10.6, and platelet count trended down to 465,000/mm^3^ (Figure 3).

The patient had clinically improved significantly and was discharged home on two weeks of the oral antibiotics (ciprofloxacin and metronidazole) course. Repeat blood work at outpatient follow-up showed complete normalization of platelet count.

## Discussion

Thrombocytosis is described as a platelet count above 450,000/mm^3^ in adults [4]. Thrombocytosis is first noticed as an incidental abnormality upon complete blood count examination in most of the cases. Thrombocytosis is mainly of two types as primary or secondary thrombocytosis. Primary thrombocytosis is caused by alterations targeting the hematopoietic cells (clonal process). Secondary thrombocytosis also called “reactive thrombocytosis” occurs in the presence of an external cause, such as chronic inflammation, cancer, iron deficiency, drug induced or rebound after splenectomy. Reactive thrombocytosis is more frequent than primary thrombocytosis and rarely causes complications on its own [5]. However, in the presence of other risk factors for bleeding or thromboembolic phenomena, it can lead to further complications.

Drugs causing thrombocytosis include vincristine, epinephrine, all-trans-retinoic acid, beta-lactam antibiotics, cytokines, and growth factors [6-7]. The increase in IL-6, other cytokines and catecholamines is suggested as a possible etiology in several reactive thrombocytosis cases [6]. However, the mechanisms are not clearly understood in drug-induced thrombocytosis. Similarly, ertapenem-induced thrombocytosis is a rare yet poorly understood phenomenon. The diagnosis of such DIT is difficult in the presence of any conditions or disease states in which thrombocytosis has been noted to occur. Parry, et al. [8] suggested that an acute-phase reaction is probably an indivisible component of an infectious process in a patient. DIT in human subjects is not substantially studied. Therefore, gauging the complications of drug-induced thrombocytosis is challenging.

Generally, DIT is a self-limited process which resolves with the discontinuation of inciting drug; in our patient’s case, it was ertapenem. The risk of thrombotic complications with any reactive thrombocytosis is low (1.6%) and has been related to the co-existence of other risk factors, such as in the postoperative setting or the presence of underlying malignancy [5]. Drug-induced reactive thrombocytosis has a small risk of bleeding, as there are no platelet function abnormalities usually seen in clonal thrombocytosis. The low risk of bleeding can also be explained by the relative deficiency of von Willebrand factor (vWF), rather than absolute deficiency, with enough free vWF multimers to keep homeostasis. In DIT, the vWF abnormalities last only for a limited period and are reversible with the normalization of the platelet count [9]. Due to the low risk of thrombotic and hemorrhagic consequences, no antiplatelet therapy is recommended for DIT.

## Conclusions

This case emphasizes a rare adverse effect of ertapenem-induced thrombocytosis, which can be problematic if left unchecked. Therefore, any patient with thrombocytosis should be assessed with a careful and detailed history of presenting illness, comorbid conditions, medications, other hematologic parameters, and past platelet counts.
